# Metal toxicokinetics and metal-driven damage to the gut of the ground beetle *Pterostichus oblongopunctatus*

**DOI:** 10.1007/s11356-016-7412-8

**Published:** 2016-08-19

**Authors:** Agnieszka J. Bednarska, Ryszard Laskowski, Elżbieta Pyza, Danuta Semik, Zuzanna Świątek, Olga Woźnicka

**Affiliations:** 1 Institute of Nature Conservation, Polish Academy of Sciences, Mickiewicza 33, 31-120 Kraków, Poland; 2 Institute of Environmental Sciences, Jagiellonian University, Gronostajowa 7, 30-387 Kraków, Poland; 3 Institute of Zoology, Jagiellonian University, Gronostajowa 9, 30-387 Kraków, Poland

**Keywords:** Trace metals, Cadmium, Nickel, Zinc, Insects, Invertebrates, Intestine, Cells, Physiology

## Abstract

Toxicokinetics makes up the background for predicting concentrations of chemicals in organisms and, thus, ecological risk assessment. However, physiological and toxicological mechanisms behind toxicokinetics of particular chemicals are purely understood. The commonly used one-compartment model has been challenged recently, showing that in the case of metals it does not describe the pattern observed in terrestrial invertebrates exposed to highly contaminated food. We hypothesised that the main mechanism shaping toxicokinetics of metals in invertebrates at high exposure concentrations in food is the cellular damage to the gut epithelial cells. Gut damage should result in decreased metal assimilation rate, while shedding the dead cells - in increased elimination rate. We performed a typical toxicokinetic experiment, feeding the ground beetles *Pterostichus oblongopunctatus* food contaminated with Cd, Ni or Zn at 40 mM kg^−1^ for 28 days, followed by a depuration period of 14 days on uncontaminated food. The male beetles were sampled throughout the experiment for body metal concentrations and histopathological examinations of the midgut. All metals exhibited a complex pattern of internal concentrations over time, with an initial rapid increase followed by a decrease and fluctuating concentrations during further metal exposure. Histopathological studies showed massive damage to the midgut epithelium, with marked differences between the metals. Cd appeared the most toxic and caused immediate midgut cell degeneration. The effects of Ni were more gradual and pronounced after at least 1 week of exposure. Zn also caused extensive degeneration in the gut epithelium but its effects were the weakest among the studied metals.

## Introduction

Toxicokinetics has been the central issue of toxicology and ecotoxicology for many years. It defines the transfer of toxic chemicals between an organism and its environment. The knowledge about toxicokinetic properties of a substance is crucial for understanding its accumulation in an organism and is the first step in predicting its potential toxic effects. Toxicokinetics of trace metals, such as zinc, copper, cadmium or nickel, is particularly interesting as metals cannot be degraded by an organism and the only way to regulate their internal body concentrations is through uptake, immobilisation and depuration. Hence, metal assimilation and elimination rates define the accumulation of metals in an organism and their ultimate equilibrium concentrations, if these exist. Trace metals have, in general terms, similar physicochemical properties so they can be expected to also have similar toxicokinetics. On the other hand, body concentrations of nutritional metals (e.g. Zn, Cu) have to be maintained within certain physiological limits to fulfil all the organism’s needs in this respect. Meeting these requirements means that, under limiting environmental concentrations, a metal has to be actively assimilated and sequestered by an organism, while at high external concentrations it has to be eliminated effectively from the body to avoid potential toxic effects. In contrast, under normal environmental conditions xenobiotic metals (e.g. Pb or Cd) do not need to be regulated that effectively as they are neither necessary for any physiological processes nor usually present in the environment at dangerously high concentrations. It has been thus hypothesised that the regulation of body metal concentrations should be more efficient for nutritional metals than xenobiotics. Indeed, this seems to be confirmed by some studies (e.g. Bednarska et al. 2015; Kramarz 1999a, b; Spurgeon and Hopkin 1999), but physiological mechanisms that might be responsible for metal level regulation in general, and for the differences between nutritional and xenobiotic metals in particular, are unknown. In their recent theoretical work Argasinski et al. (2012) postulated that internal metal concentration in invertebrates, at least when a metal is consumed with food at high concentration, can simply be the side-effect of metal accumulation in the gut epithelial cells, and the resulting toxicity to the cells being the first line of defence against excessive body concentrations of metals and their toxicity in insects (Rost-Roszkowska et al. 2008). The damage to the gut cells should depend on both the concentration of a metal and its inherent toxicity. Higher metal concentrations, especially if a metal is very toxic, should cause extensive damage to the gut epithelium, resulting in an increased shedding of dead and/or dysfunctional cells. In a toxicokinetic study this would be observed as an increased elimination rate due to excretion of the shed cells, and a decrease in assimilation rate due to the damage of the gut epithelium. The theoretical model formulated by Argasinski et al. (2012) allowed for explaining the specific phenomenon observed in some studies on metal toxicokinetics in invertebrates, up to then considered an artefact, namely an abrupt increase of internal metal concentration upon exposure to metal-contaminated food, followed by a gradual concentration decrease, even if an animal was still exposed to the contaminated food (e.g. Bednarska et al. 2011; Lagisz et al. 2005; Neuhauser et al. 1995). Regardless of earlier attempts to model such unexpected toxicokinetics in carabids (Bednarska et al. 2011; Laskowski et al. 2010), the models were purely phenomenological as no data on metal toxicity to gut epithelial cells were available for their parametrisation.

**Table 1:** Nominal and actual concentrations of metals (Cd, Ni, Zn) in food and in beetles before metal exposure (*C*_*I0*_), estimated toxicokinetic parameters (*k*_*A*_ – assimilation rate constant, *k*_*E*_ – elimination rate constant) and *R* ^*2*^ for the classic one-compartment model, bioaccumulation factor (*BAF = k*_*A*_ */k*_*E*_), equilibrium concentration, i.e. the concentration expected in beetles at a specific external metal concentration in food (*C*_*E*_ ) at t_∞_ (*C*_*eq*_ *= C*_*E*_ *k*_*A*_ */k*_*E*_) and survival (%) for *Pterostichus oblongopunctatus* exposed to metal-contaminated food. Metal concentrations are reported as means ± SD, estimated toxicokinetic parameters are given with 95 % confidence intervals

*Metal*	*Metal concentration in contaminated food*		*Metal concentration in control food*	*C*_*I0*_	*k*_*A*_	*k*_*E*_	*R* ^*2*^	*BAF*	*C*_*eq*_	*Survival*
	*Nominal*	*Actual*								
	[mM kg^−1^]		[mM kg^−1^]	[mM kg^−1^]	[day^−1^]	[day^−1^]	%		[mM kg^−1^]	%
Cd	40	49.9 ± 5.4	0.002 ± 5.4 × 10^−5^	1.21 × 10^−3^ ± 1.58 × 10^−3^	0.038 (0.013–0.063)	1.32 (0.445–2.185)	0.029	20.8	1.44	89
Ni	40	41.53 ± 1.1	0.29^*^	0.23 ± 0.195	0.033 (0.015–0.051)	0.70 (0.315–1.085)	0.046	15.0	1.95	93
Zn	40	40.81 ± 1.0	4.45 ± 0.122	1.17 ± 0.029	0.079 (0.013–0.144)	1.64 (0.290–2.991)	0.048	8.3	1.96	78

^*^– only one result out of three samples is reported; the remaining two samples were below the detection limit

Traditionally metal toxicokinetics in invertebrates is described by a classic one-compartment model with metal uptake determined by its concentration in food and the assimilation rate constant *k*_*A*_, which is supposed to be specific for a metal and a species. Similarly, metal excess is expected to be eliminated from the body at a metal- and species-specific elimination rate constant *k*_*E*_. For example, Janssen et al. (1991) showed that cadmium uptake (i.e., the amount of the metal ingested by an organism per unit time) and elimination rates differed substantially between species representing different taxonomic positions. Similar conclusions can be reached when comparing metal toxicokinetics of Cd and Zn in the carabid beetle *Poecilus cupreus* (Kramarz 1999a) and the centipede *Lithobius mutabilis* (Kramarz 1999b). More recently Bednarska et al. (2015) showed, however, that although different metals indeed differ in their toxicokinetics, there is nothing like metal-specific assimilation or elimination rates. After testing the toxicokinetic parameters for a range of exposure concentrations of Zn and Cd in crickets *Gryllus assimilis*, the authors concluded that both the assimilation and the elimination rate constants can change depending on the exposure concentration. This finding confirms indirectly the idea formulated by Argasinski et al. (2012) that internal metal concentration in invertebrates can be, at least to a certain extent, a direct outcome of metal toxicity to the gut epithelial cells.

The aim of the present study was to test the hypothesis that direct metal toxicity to the gut epithelial cells is the important physiological mechanism determining the toxicokinetics of metals consumed with highly contaminated food. We performed a typical toxicokinetic experiment on the ground beetle *Pterostichus oblongopunctatus* (Coleoptera: Carabidae) exposed to food contaminated with Cd, Ni or Zn, in which we followed not only internal metal concentrations in the male beetles but also morphological changes in the midgut epithelium cells by light and electron microscopies. Additionally, the TUNEL reaction was used to detect apoptosis. To our knowledge, we show for the first time the dynamics of cellular damage in the gut epithelium during the toxicokinetics experiment and relate them to metal exposure and metal concentrations in the beetles.

## Materials and methods

### Research area and beetle sampling

Adult ground beetles *P. oblongopunctatus* were collected with pitfall traps (plastic cups, ca. 200 ml) from a Scots pine forest in an unpolluted area near Pilica (50°28’N 19°39’E), southern Poland, in April and May 2011 and 2013. Only male beetles, collected from the same area were used in the study, as it was probable that at least some of the captured females were already fertilised (Brunsting 1981), which is known to influence the metabolism (Chaabane et al. 1999). The beetles were kept for up to four weeks in a climatic chamber at 20 °C and 75 % relative humidity (RH) under a light:dark regime 16 h:8 h, in plastic boxes (ca. 1000 ml, with perforated lid), 12 to 16 males in each. The boxes were filled to approximately 1 cm with moistened peat, and a piece of bark picked up from the sampling area was placed in each box to provide a shelter for the beetles. The beetles were fed ad libitum, three times a week, with artificial food made of ground mealworms (*Tenebrio molitor*) mixed with ground apple (Nestlé, Gerber) in a ratio of 7:3 (w:w) with ca. 0.8 g sodium benzoate (C_7_H_5_NaO_2_; Fluka, Germany) per kg of food as a preservative. Before starting the toxicokinetics experiment, the animals were weighed to the nearest 0.0001 g (Radwag AS/C/2, Poland) and placed individually in 30-ml plastic boxes filled to ca. 1/4 with moistened sand (Grudzeń Las Sp. z o. o., Poland). In each box a piece of bark was placed to provide a shelter for the beetles.

Cadmium chloride (CdCl_2_ × 2.5 H_2_O, POCH, Poland), zinc chloride (ZnCl_2_, Merk, Germany), or nickel chloride (NiCl_2_ × 6H_2_O, Eurochem BGD, Poland) were added to the dried food as aqueous solutions at 40 mM kg^−1^ dry food, and redistilled water was added to the control food. After bringing to 75 % of the original moisture, the food was frozen in small portions at −20 °C. The animals were fed ad libitum every second day with a new portion of unfrozen food. Remains of the old food were removed each time during feeding to keep the boxes clean.

### Experimental design

Only males were used in two toxicokinetics experiments run in 2011 and 2013 to eliminate potential sex effects. Beetles collected in 2013 were used for both chemical and histopathological analysis, whereas those collected in 2011 were used only for histological analysis. The beetles were randomly allocated to treatments (Cd, Ni or Zn) and were fed metal-contaminated food for 28 days (uptake phase), and uncontaminated food for another 14 days (decontamination phase). The experimental design was based on earlier studies on carabids in which it was found that a 28-day period is long enough to reach an equilibrium body metal level (if such a level exists for a particular metal) and 14 days are sufficient to decontaminate totally or excrete at least part of the assimilated metal, depending on the metal used (Bednarska et al. 2011; Lagisz et al. 2005; Kramarz 1999a). For chemical analyses, the beetles were sampled before starting the exposure (day 0) and after 0.5, 1, 1.5, 2, 4, 6, 8, 12, 16, 20, 24, 28, 28.5, 29, 29.5, 30, 32, 34, 38 and 42 days since the start of experiment. Until the 20th day five individuals were sampled from each metal treatment but later the number of sampled beetles had to be reduced to four or three because of the increased mortality. The beetles sampled for chemical analyses were kept individually in empty boxes for 24 h to empty the gut content, washed in deionised water to remove all remains of food and/or sand from their body surface, weighed to the nearest 0.0001 g (Radwag AS/C/2, Poland) and killed by freezing at −20 °C. For histopathological analyses, five individuals were additionally sampled at days 0, 1, 2, 16, 28 (last day of metal exposure) and 42 (last day of the experiment, after two weeks of depuration) in 2013. The sampling schedule for histopathological analysis in the experiment in 2011 was the same as for the chemical analysis in 2013, with the only differences that the beetles were sampled at days 36 and 40 instead of 38 and 42 in 2013 and 2–3 individuals instead of 5 were sampled in 2011. The beetles (100 individuals from 2013 and 118 from 2011) were sacrificed by decapitation immediately after sampling, their midguts were dissected and used for light microscopy and transmission electron microscopy (TEM) analysis of the gut epithelium and TUNEL reaction. The TUNEL detection of apoptotic cells was repeated twice. Animals that died over the course of the experiment were excluded from the analyses.

### Chemical analyses

The frozen beetles were dried at 105 °C for 24 h, weighed to the nearest 0.0001 g (Radwag AS/C/2, Poland) and digested in 2 ml of a 4:1 mixture of HNO_3_ (65 %, INSTRA-ANALYSED, Baker, Germany) and HClO_4_ (65 %, Ultranal POCh, Poland) at temperatures increasing gradually from 20 °C to 300 °C. After complete digestion, the excess of acids was evaporated and the samples were resuspended to 3 ml with 0.2 % HNO_3_. Because of the large number of individuals, the beetles were analysed in four series. Three samples of food from each treatment, ca. 0.5 g dry weight, were digested in 10 ml of a 4:1 mixture of nitric and perchloric acids, evaporated and then resuspended to 10 ml with 0.2 % HNO_3_. To determine the analytical precision, three blanks and three samples of reference materials – fish liver (*Certified Reference Material Dolt-4 Dogfish Liver*, National Research Council of Canada) and sea lettuce (*Certified Reference Material BCR® no. 279 Ulva lactuca*, Institute for Reference Materials and Measurements, Belgium) – were run with the beetles and food samples, respectively. Ni concentrations were analysed with a graphite furnace atomic absorption spectrophotometer (Perkin-Elmer AAnalyst 800), Zn with flame–atomic absorption spectrophotometer (Perkin-Elmer model AAnalyst 200) and both methods were used for Cd concentrations depending on the concentration in the samples. The concentrations of metals were expressed in mM kg^−1^ dry weight (dw).

The average concentrations of Cd and Zn measured in the reference material for food analysis were ca. 7 % lower than the certified values. The certified value for Ni in sea lettuce is not given by the producer. The certified and measured concentrations in the reference material for beetles were, respectively, 24.3 ± 0.8 and 18.1 ± 8 mg kg^−1^ for Cd, and 116 ± 6 mg kg^−1^ and 69.2 ± 51 mg kg^−1^ for Zn. Ni concentrations in the fish liver was not measured due to too low concentration of this metal for the sample sizes used (the beetles). The small samples size, ca. 120 mg, which is substantially less than the minimum sample size (250 mg) recommended by the producer, was the most probable source of the high variance in Cd and Zn concentrations measured in the reference material. We used ca. 120 mg to assure similar digestion and analytical procedure as had to be used for the beetles. Metal concentrations measured in the food and beetles were not corrected for recovery.

### Light microscopy

#### Midgut histopathology

The guts of the beetles were removed, the midguts isolated and fixed in a mixture of 85 % ethanol, 10 % paraformaldehyde and 5 % of acetic acid for 24 h at 10 °C. After fixation the tissues were dehydrated in graded concentrations of ethanol series, cleared in xylene and transferred to a mixture of xylene and paraplast (Sigma, USA) first, next to paraplast alone, and finally embedded in paraplast. After polymerisation, 7 μm longitudinal sections of midguts (10 preparations per animal) were cut (Leica Jung Multicat 2045 microtome) and treated with xylene to remove paraplast, hydrated in ethanol series and stained with haematoxylin and eosine. Finally, they were again dehydrated, cleared in xylene and embedded in Canada balsam. Three sections of the midgut epithelium of each beetle were photographed (LM AXIOSCOP Zeiss, lens 40×) and the height of the epithelium was measured three times per image in different regions of the epithelium using ImageJ software. For statistical analysis, the average epithelium height of all the measurements taken in a single beetle was taken as a replicate.

#### Detection of apoptotic cells

The midgut sections were treated with xylene twice for 5 min each to remove paraplast, hydrated in ethanol (twice in 100 % for 5 min each and 5 min in 96 %), and rinsed in distilled water (5 min). Next, they were washed in phosphate buffer saline with Tween 20 (PBS-Tween 20) (5 min) and treated for 20 min at 37 °C with DNase I (DNase I, AMPD1-1KT, Sigma) (5 μg/ml). After two washes in PBS-Tween 20 (5 min each) endogenous peroxidase was blocked for 10 min with 3 % H_2_O_2_ in PBS, pH = 7. Next, after three washes (2 min each) in PBS-Tween 20 sections were incubated with TUNEL Reaction Mixture (Roche Diagnostic, Germany) at 37 °C. The TUNEL assay was performed according the protocol obtained from the IHCWORLD home page (http://www.ihcworld.com_ protocols/apoptosis/tunel_enzyme.htm) and already published methods (Gavrieli et al. 1992; Charriaut-Marlangue and Ben-Ari 1995). In this method sections were pre-incubated for 10 min with TdT (terminal transferase) Reaction Buffer and incubated in TdT Reaction Mixture (TdT and Biotin-16-dUTP in TdT Reaction Buffer) for 2 h at 37 °C. To terminate the reaction stop wash buffer was used (10 min). Next sections were rinsed in PBS-Tween 20 (3 × 2 min) and left for 30 min in Streptavidin-HRP in PBS-Tween 20 at room temperature. After rinsing in PBS-Tween 20 (3 × 2 min) they were treated with DAB (0.05 %) for 1–3 min, rinse in tap water and counterstained with haematoxylin. After rinsing in tap water (5 min) sections were coversliped with glycergel mounting medium. As a positive control, TUNEL assay was performed on sections obtained from young rat liver. As a negative control, the midgut sections were incubated with TUNEL Reaction Mixture without TdT.

### Transmission electron microscopy

The midguts (60 individuals collected in 2011) were fixed for electron microscopy in 2.5 % paraformaldehyde and 2.5 % glutaraldehyde in 0.1 M cacodylate buffer for 2 h and next in 2 % OsO_4_ in veronal buffer for 2 h at room temperature. Next, fixation tissues were dehydrated in ethanol series and twice in propylene oxide. They were embedded in Poly/bed 812 (Polysciences) resin and, after polymerisation, 70 nm ultrathin sections were cut with a Reichert Ultracut and collected on single slot grids coated with a formvar film. The sections were stained with uranyl acetate and lead citrate. They were viewed and photographed with a transmission electron microscope (TEM) JEOL 100 SX for ultrastructural analysis of the midgut cells.

### Statistical analysis

The pattern of changes in internal metal concentrations (*C*_*int*_) in time (*t*) was shown as means with standard errors. The one-compartment toxicokinetics model was fitted to metal concentrations measured in individual beetles to determine to what extent the pattern can be described with this classic model. We used the equations given by Skip et al. (2014):

for the uptake phase (*t* < *t*_*c*_):

$$ {C}_I(t)={C}_{I0}\cdot {e}^{-{k}_Et}+{C}_{Eu}\frac{k_A}{k_E}\left(1-{e}^{-{k}_Et}\right), $$

and for the decontamination phase (*t* > *t*_*c*_):

$$ {C}_I(t)={C}_{Itc}\cdot {e}^{-{k}_E\cdot \left(t-{t}_c\right)}+{C}_{Ed}\frac{k_A}{k_E}\left(1-{e}^{-{k}_E\left(t-{t}_c\right)}\right), $$

where

$$ {C}_{I{t}_c}={C}_{I0}\cdot {e}^{-{k}_E\cdot {t}_c}+{C}_{Eu}\frac{k_A}{k_E}\left(1-{e}^{-{k}_E\cdot {t}_c}\right). $$

where: *k*_*A*_ – assimilation rate constant [day^−1^], *k*_*E*_ – elimination rate constant [day^−1^], *t*_*c*_ – the time of changing the food from contaminated to uncontaminated (here: 28th day of the experiment) [days], *C*_*0*_ – initial body metal concentration at *t* = 0, given in the model explicitly as the average concentration of a metal measured in five individuals before starting the exposure (Day 0) [mg kg^−1^ dw], *C*_*Eu*_ and *C*_*E*d_ – the exposure concentration in food in the uptake and decontamination phase, respectively [mg kg^−1^ dw], *e* – the base of natural logarithm. Kinetic parameters *k*_*A*_ and *k*_*E*_ for each metal treatment were obtained by simultaneous fitting the equations to the data from both experimental phases using the least squares method (GraphPad Prism by GraphPad Software, Inc.). All the parameters were checked for significance using 95 % confidence intervals. The confidence intervals around the estimated parameters were also used to compare the treatments. The estimated toxicokinetic parameters were used to calculate bioaccumulation factors (*BAF* = *k*_*A*_/*k*_*E*_) and theoretical equilibrium metal concentrations in the beetles at *t*_*∞*_, *C*_*eq*_ *= C*_*Eu*_∙(*k*_*A*_/*k*_*E*_).

In 2011, because of the limited number of available animals, only 2–3 beetles could be collected on each sampling day per treatment for light microscopy. As this is insufficient for adequate statistics, these preparations were described only qualitatively, and formal statistical analyses were performed on beetles from the 2013 experiment, with 5 beetles per treatment sampled on each occasion (100 individuals in total).

The metal effect on the midgut epithelium height, as measured on histological preparations from the 2013 experiment, was analysed separately for each metal with one-way ANOVA. If ANOVA indicated an overall effect significant at *p* ≤ 0.05, it was followed by a Fisher LSD test for post-hoc planned comparisons, contrasting each day against day 0, i.e. the epithelium height before metal exposure. The results were presented graphically as arithmetic means with standard deviations, and days differing from day 0 at p ≤ 0.05 were marked accordingly.

## Results

The actual metal concentrations in food were in good agreement with the nominal ones (Tab. 1). Despite the high metal concentrations used in the experiment, the survivorship of the beetles was satisfactory, with 78 % to 93 % individuals surviving until the end of the experiment (Tab. 1).

### Metal concentrations in beetles and toxicokinetics

**Fig. 1:**
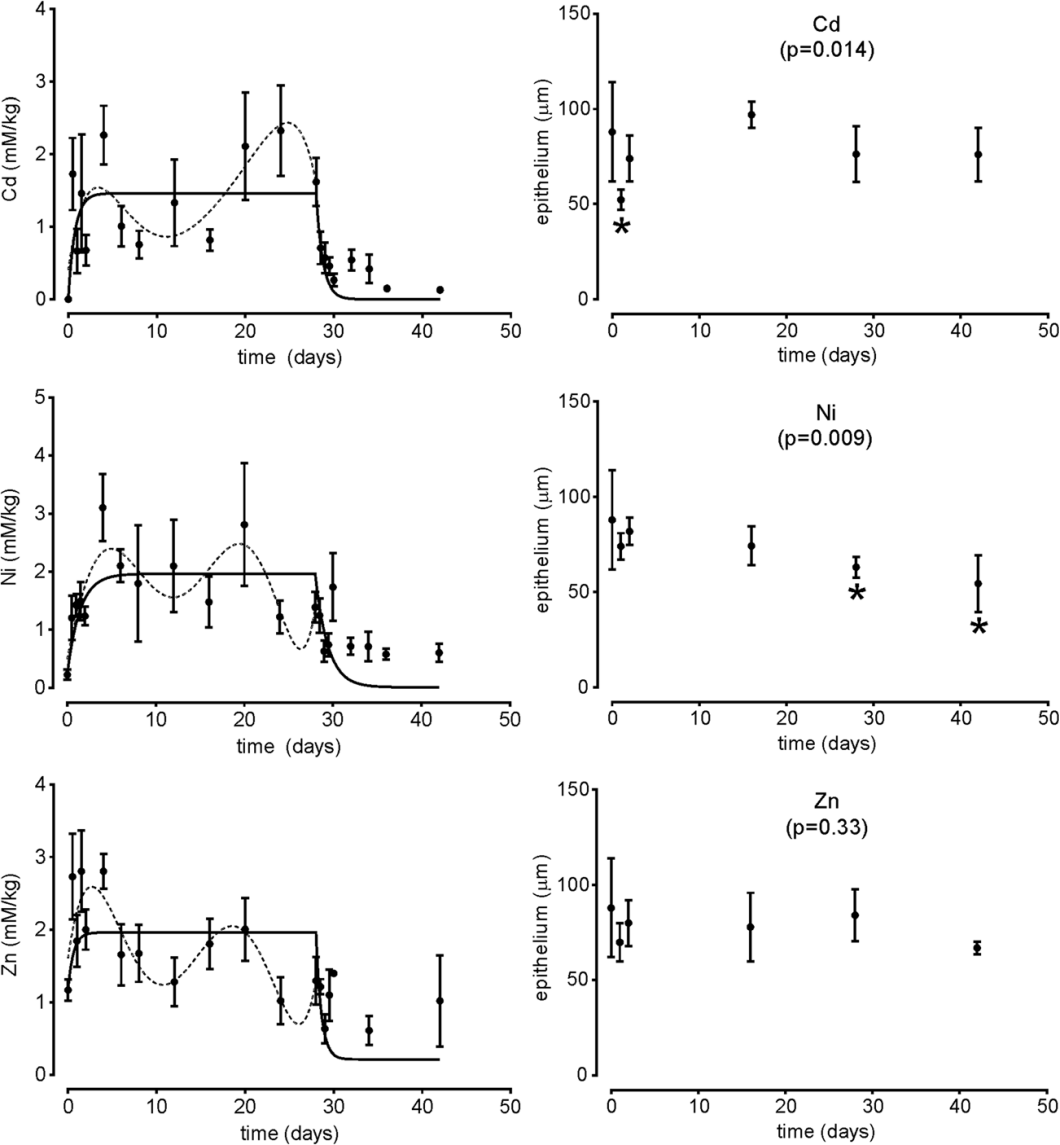
Toxicokinetics of cadmium (Cd), nickel (Ni) and zinc (Zn) (left-hand column) and midgut epithelium height (right-hand column) in the ground beetle *Pterostichus oblongopunctatus* fed artificial food contaminated with metals at nominal concentration of 40 mM kg^−1^ (for actual concentrations, see Table 1). Toxicokinetics: points – body concentration of a metal; whiskers – standard error of the mean; solid line – fitted one-compartment model; broken line – polynomial showing the observed temporal fluctuations in metal concentrations. Epithelium: points – mean height of the epithelium; whiskers – standard deviation; asterisks below whiskers denote statistically significant difference (*p* ≤ 0.05) in comparison to day 0; overall significance level (ANOVA) for metal effect on the epithelium height is given below the metal symbol

The initial average internal body concentration of cadmium was 0.0012 mM kg^−1^ dw. Within 12 h the concentration reached 1.73 mM kg^−1^, and the highest average concentration occurred at days 4 (2.26 mM kg^−1^) and 24 (2.32 mM kg^−1^). Between these two peaks, the concentration dropped substantially to 0.75 mM kg^−1^ at the 8th day of exposure. After switching the animals to uncontaminated food, the average body Cd concentration decreased to 0.13 mM kg^−1^ at the end of the experiment (Fig. 1). The initial concentration of nickel in the beetles was 0.23 mM kg^−1^ dw. Similar to the Cd treatment, the maximum Ni concentration was detected at the 4th day (3.11 mM kg^−1^ dw), then decreased to 1.48 mM kg^−1^ and raised again at the 20th day to 2.81 mM kg^−1^. After 2 weeks of decontamination, the concentration dropped to 0.6 mM kg^−1^. The average initial Zn body concentration at day 0 was 1.17 mM kg^−1^, and the highest average concentration, 2.81 mM kg^−1^, was reached after 1.5 days of exposure and at the 4th day. Then, the concentration gradually decreased to 1.28 mM kg^−1^ at the 12th day to increase again up to 2.01 mM kg^−1^ at the 20th day. After transferring the beetles to uncontaminated food, a further decline in the body zinc concentration was observed to ca. 1.02 mM kg^−1^ at the end of the experiment. In all cases the highest concentrations of metals were reached at the 4th day of exposure and later between days 20 and 24. In between, concentrations always decreased substantially, reaching their lowest levels between days 8 and 16 (Tab. 1, Fig. 1).

Although concentrations of all three metals apparently fluctuated over time, the classic one-compartment model could be fitted to the data (Fig. 1) but explained only 20.8, 15.0 and 8.3 % of the total variance for Cd, Ni and Zn, respectively (Tab. 1), indicating that it does not describe the toxicokinetics of the metals well. Indeed, the polynomial curves (sixth order) superimposed on the graphs, although with no analytical meaning, better fit the observed temporal fluctuations of metal concentrations, suggesting dynamic changes in metal uptake and excretion during metal exposure (Fig. 1). We discuss later these fluctuations in relation to the observed changes in the intestine and natural physiological processes.

None of the confidence intervals for *k*_*A*_ or *k*_*E*_ covered 0, indicating that despite the rather poor fit, the models are statistically significant and the parameters estimated by the one-compartment model are trustworthy. The estimated toxicokinetic parameters (Tab. 1) did not differ between the metals, as indicated by the overlapping confidence intervals. The lowest assimilation rate constant, *k*_*A*_, was found for Ni (0.033 day^−1^) and was almost identical with that for Cd (0.038 day^−1^) and ca. two times lower than for Zn (0.079 day^−1^). The elimination rate constants, *k*_*E*_, ranged from 0.7 for Ni, through 1.32 for Cd and 1.64 for Zn. The resulting bioaccumulation factor (BAF) was the lowest for Cd (0.029), and those for Ni and Zn were nearly identical: 0.047 and 0.048, respectively. The calculated equilibrium concentrations (*C*_*eq*_), assuming infinite exposure to the actual experimental concentrations of the metals in food, were: 1.44 mM kg^−1^ for Cd, 1.95 mM kg^−1^ for Ni, and 1.96 mM kg^−1^ for Zn (Tab. 1).

### Midgut histopathology

**Fig. 2:**
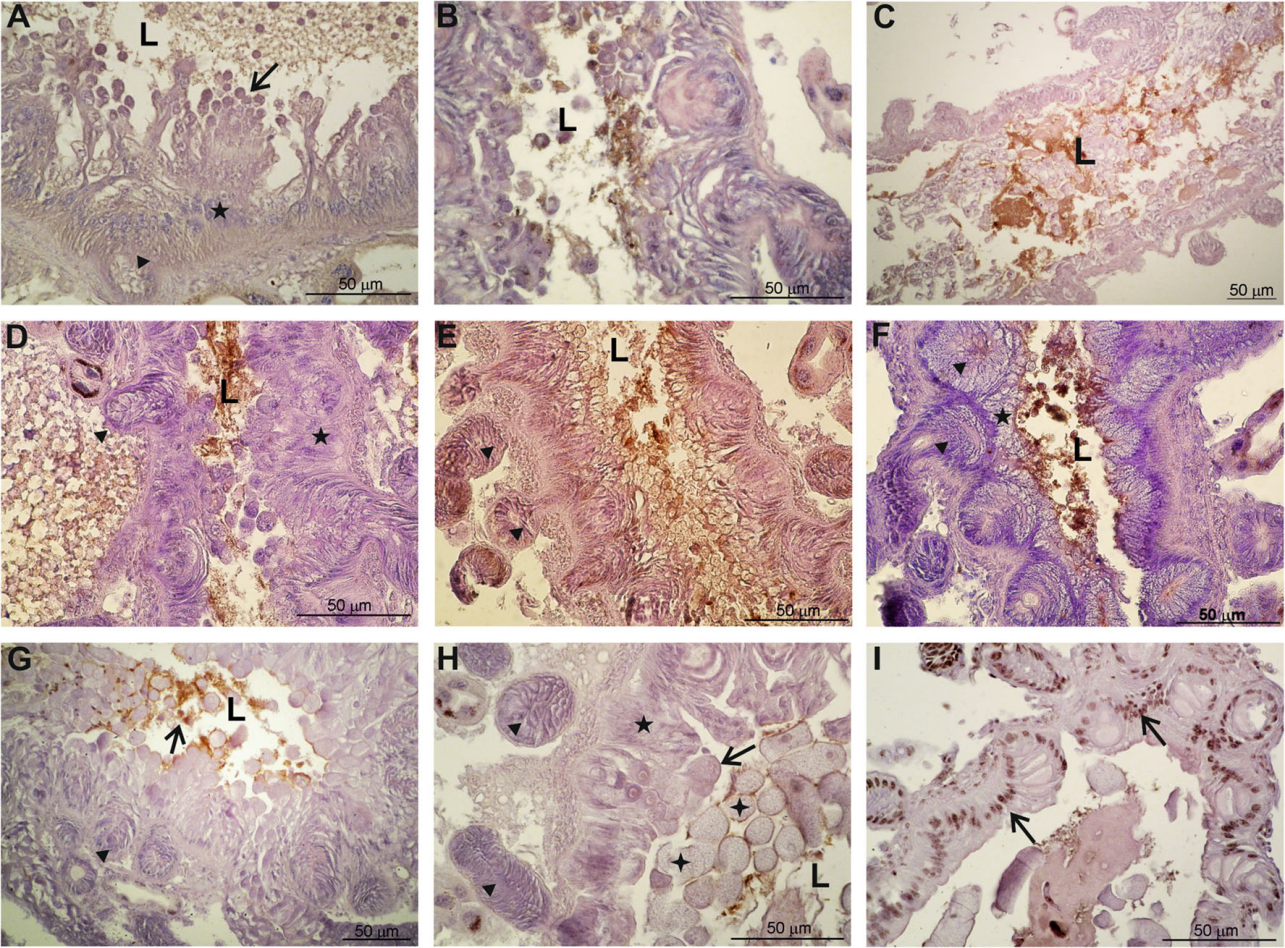
Light microscope longitudinal sections of the beetle *Pterostichus oblongopunctatus* midgut in control and exposed beetles treated with Cd, Ni and Zn. L – the midgut lumen. Arrowheads indicate regenerative crypts. A to I – sections were processed according to the TUNEL method to detect apoptotic cells and counterstained with haematoxylin. A: midgut of a control beetle with epithelial (star) and digestive cells (arrow). B, C: 1st and 2nd day of Cd exposure. The epithelium is very low and cell and cell debris are observed in the midgut lumen. D, E, F: 8th, 12th and 34th day of the experiment with Ni. Epithelial cells degenerate after 8 days of exposure (D). The midgut lumen is filled with cells and cell fragments (E). After one week of detoxification, 34th day of the experiment, the midgut starts to regenerate and epithelial cells (star) are visible (F). G: 4th day of Zn exposure. Apoptotic cells (TUNEL-positive cells) are in the midgut lumen (arrow). H: 16th day of Zn exposure. The midgut contains a thin layer of epithelial cells and a few digestive cells. Portions of cells are discharged to the midgut lumen. The lumen is also filled with parasites (stars). I: 32nd day of the experiment with Zn, 4th day of detoxification. Apoptotic cells (nuclei are dark brown, arrows) are in the epithelium and in crypts

**Fig. 3:**
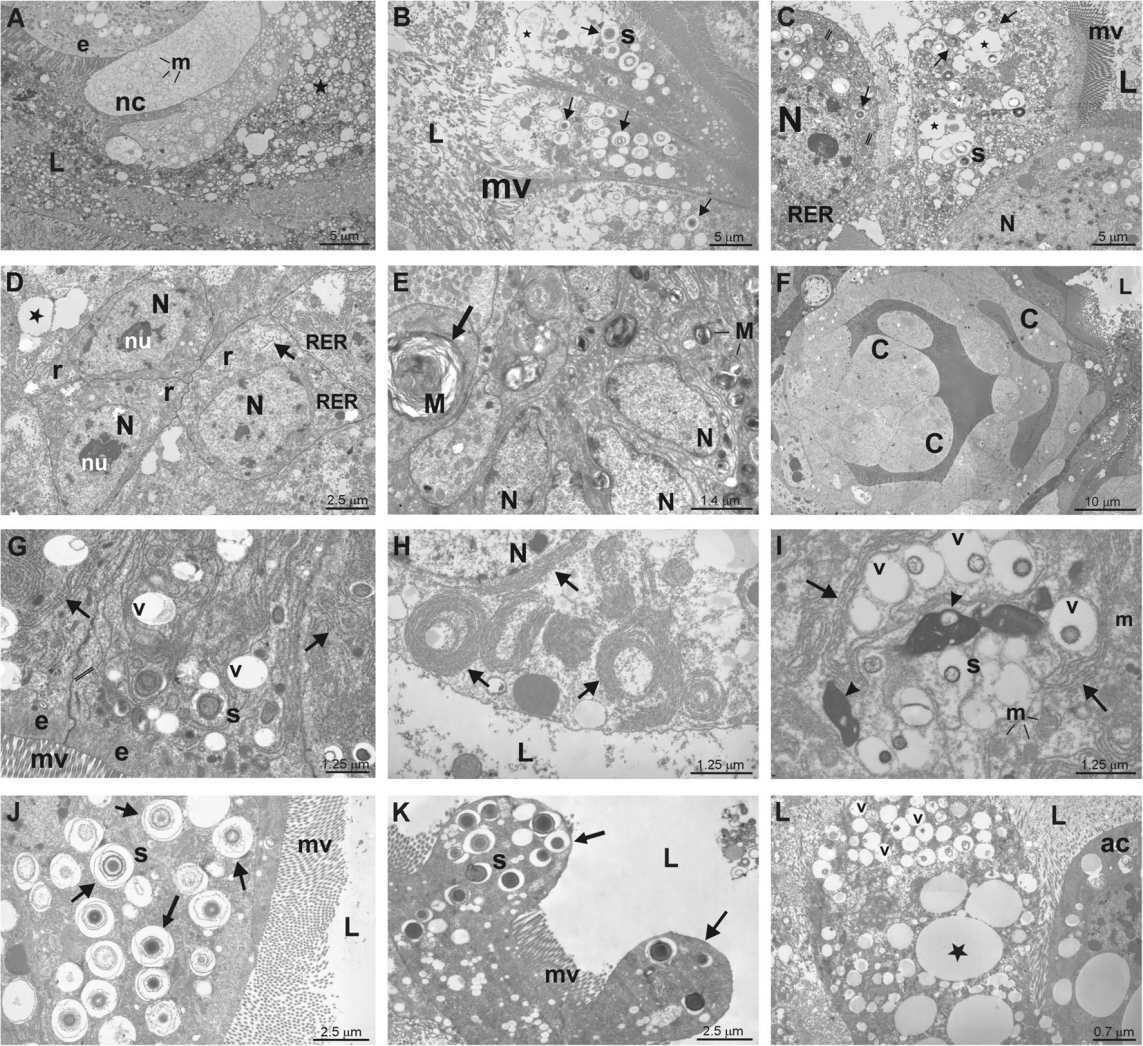
Examples of degenerative changes at transmission electron microscope (TEM) level in the midgut cells of *Pterostichus oblongopunctatus* treated with Cd, Ni or Zn. L – the midgut lumen, mv – microvilli, N – nucleus, nu – nucleolus, stars and v – vacuoles, m – mitochondria, e – epithelial cells, r – regenerative cells in crypts, RER – cisterns of rough endoplasmic reticulum, s – spherites, M – myelinoid body. A: control, epithelial cell (e) neighbours necrotic cells (nc) with swollen mitochondria (m). B: 4th day of Ni exposure, apical portions of epithelial cells containing vacuoles and Ni spherites (arrows) are discharged to the lumen. C: 2nd day of Zn exposure, epithelial cells contain numerous vacuoles and Zn spherites (s). D: 2nd day of Zn exposure, regenerative cells (r) in a crypt showing vacuoles and translucent cytoplasm (arrow). E: 16th day of Ni exposure, an arrow indicates autophagosome (M- myelinoid body, arrow). F: 20th day of Zn exposure, crypt cells (c) are swollen and epithelial cells above crypt cells are degenerated. G: 12th day of Cd exposure, cells contain numerous vacuoles (v) and spherites (s) surrounded by endoplasmatic reticulum membranes (arrows). H: 8th day of Ni exposure, endoplasmatic reticulum forms membranous structures (arrows). I: 12th day of Zn exposure, endoplasmatic reticulum membranes (arrows) surround organelles in autophagosomes, arrowheads indicates residual bodies. J: 4th day of Ni exposure, arrows indicate Ni spherites. K: 8th day of Zn exposure, the budding of epithelial cell cytoplasm with spherites (arrows). L: 8th day of Ni exposure, an epithelial cells with numerous vacuoles and apoptotic cell (ac) discharged to the midgut lumen. Magnification: A, B, C ×1800; D, L ×2800; E, G ×5

The midgut of control *P. oblongopunctatus* contained regenerative crypts and epithelial cells which release digestive enzymes by budding of apical portions of the epithelial cells to the midgut lumen (Figs. 2A, 3A). The exposure to Cd induced fast degeneration of the midgut by necrosis. Epithelial necrotic cells or their fragments were discharged to the midgut lumen after 1–2 days of exposure, and the midgut contained only degenerating epithelial cells filled with vacuoles, spherites and membranes (Fig. 3I) and regenerative crypts (Fig. 2B, C) which partly degenerated during consecutive days of the exposure. TUNEL-positive cells were not detected in the midgut of beetles exposed to Cd. The acute effect of Cd on the midgut epithelium was confirmed by a significantly lower epithelium height at the 1st day after exposure (Fig. 1). However, in the following days the epithelium height was similar to the one measured at the beginning of the experiment (day 0), probably because the remaining epithelial and crypt cells were swollen.

The exposure to Ni induced slower degeneration of the midgut than in case of Cd. At the 8th day of exposure the regenerative crypts were still present in the epithelium (Fig. 2D). At the 12th day of exposure the midgut contained mostly degenerating crypt cells and digestive cells and their fragments were observed in the midgut lumen (Fig. 2E). This progressive degeneration of the midgut, mostly by necrosis, was also confirmed by the gradual decrease of the epithelium height during the exposure (Fig. 1). On the 34th day of the experiment, after 6 days of decontamination, the first symptoms of midgut regeneration were observed (Fig. 2F). We noticed more regular crypts in the midgut and the first layer of epithelial cells. Nevertheless, the epithelium height was significantly lower than before the exposure even at the end of the experiment (day 42; Fig. 1). The overall Ni effect on the epithelium height was significant at *p* = 0.009, and the first significant difference when compared with day 0 occurred after 28 days of exposure (Fig. 1).

Zinc was less toxic to the midgut cells than Cd or Ni, and degenerative changes were observed later than in the beetles exposed to either of the latter two metals. This was confirmed by non-significant changes in the gut epithelium height throughout the experiment (*p* = 0.33, Fig. 1). After 1 day of exposure the epithelium was mostly not affected (Fig. 2G), but a few TUNEL-positive cells in the epithelium were observed. Numerous TUNEL-positive cells appeared in the outer layer of the epithelium at the 4th day of exposure, and later in the whole epithelium (20th day of exposure). At days 8–20 the gut epithelium was very low and some of crypt cells were TUNEL-positive. After 28 days of intoxication with Zn, massive degeneration of the epithelium was observed with many TUNEL-positive cells in the lumen of the midgut. However, the height of the gut epithelium measured in the beetles sampled in 2013 did not change, probably because of swelling of the remaining cells (Fig. 1). On the 32nd day of the experiment, after 4 days of decontamination, majority of cells in the regenerative crypts were TUNEL-positive (Fig. 2I). TUNEL-positive cells were detected only after the exposure to Zn but not to Cd or Ni. The epithelium was not recovered on day 40, i.e. 12 days after the termination of Zn exposure. By the end of experiment, the epithelium had an irregular structure, but its height at day 42 did not differ from day 0 (Fig. 1).

The midgut of most of the beetles, including the control, was infected by parasites from Gregarinasine. The parasites were observed in the midgut lumen and in the epithelium between the cells. The parasites were less frequent after Cd and Ni exposures but were present after two weeks of Zn exposure when the midgut contained regenerative crypts and some digestive cells but most of the epithelial cells were discharged to the gut lumen (Fig. 2H).

The examination at TEM level showed that degenerating cells of the epithelium in metal exposed beetles are filled with metal-containing spherites and other vacuoles (Fig. 3B, C, J, K, L), in contrast to the epithelial cells of the control animals that contain less vacuoles and vesicles containing digestive enzymes which are released to the midgut lumen (Fig. 3A). In the epithelium of control insects there are also degenerating cells since the midgut epithelium is in continuous renewal. The apical parts of those cells, containing spherites, were discharged to the midgut lumen (Fig. 3K) or whole cells were disintegrated and their content was released to the lumen (Fig. 3B, C). Budding portions of cells with spherites were often observed in the beetles intoxicated with Zn and Ni, while cell swelling and disintegration was seen in those exposed to Cd. In the lumen, not only fragments of cells were observed but also whole degenerated epithelial cells with apoptotic nucleus. Degenerating changes were also observed in crypt cells (Fig. 3D, E) which had vacuoles, translucent cytoplasm and autophagy-like structures in the cytoplasm. In some cells membranous structures, probably formed from endoplasmatic reticulum membranes (Rost-Roszkowska et al. 2010) which are similar to autophagosomes, were observed in the epithelial cells (Fig. 3E, G, H, I). After 20 days of exposure, cells in regenerative pouches were often swollen and the crypts disintegrated (Fig. 3F). Cells were dispersed in crypts.

## Discussion

To compare the toxicokinetics of different metals, parameters for toxicokinetic models have to be tested under exposure to an identical amount of ions for different metals, as the free metal ion is a single entity that can be taken up by an organism and interacts with cells in the alimentary tract (Bednarska et al. 2015). Thus, the important problem to tackle when designing the experiments on Cd, Ni and Zn was choosing the exposure concentrations that would allow for comparison of the toxicokinetics and effects of the three metals that differ in their natural concentrations in the environment. In uncontaminated soils, concentrations of Cd range between 0.06 and 1.1 mg kg^−1^ (0.0005–0.01 mM kg^−1^), those of Ni are 0.2 to 450 mg kg^−1^ (0.003–7.7 mM kg^−1^), and of Zn 10 to 300 mg kg^−1^ (0.15–4.6 mM kg^−1^) (Kabata-Pendias and Mukherjee 2007). The highest reported concentrations in soil have been recorded around metal smelters, and for Cd reach over 1700 mg kg^−1^ (15 mM kg^−1^), for Ni up to 26,000 mg kg^−1^ (443 mM kg^−1^), and for Zn over 10,000 mg kg^−1^ (153 mM kg^−1^) (Kabata-Pendias and Mukherjee 2007). Potential food for ground beetles, such as plants and small invertebrates, usually has lower concentrations but some extreme cases have also been reported, such as plants with Cd up to 560 mg kg^−1^ (4.98 mM kg^−1^), Ni up to 6000 mg kg^−1^ (102 mM kg^−1^), and Zn reaching 10,000 mg kg^−1^ (153 mM kg^−1^) (Kabata-Pendias and Mukherjee 2007). The reported data show that, at least theoretically, beetles can be exposed to concentrations of Ni and Zn even higher than those used in our study (ca. 40 mM kg^−1^). Cadmium concentration used in this study was substantially higher than the highest recorded concentrations of this metal in soil or plants. However, the aim of the study required using equimolar concentrations of all metals, high enough to exert effects postulated by Argasinski et al. (2012). According to the model by Argasinski et al. (2012), the specific toxicokinetic pattern with a peak metal body concentration within the first 1–4 days of exposure, followed by a concentration decrease even if an animal is still exposed to contaminated food, occurs approximately when more than 50 % of the gut epithelial cells are damaged. Such pattern was observed by Bednarska et al. (2011) in *P. oblongopunctatus* exposed to Ni in food at the concentration of 2500 mg kg^−1^ (42.6 mM kg^−1^) and predictions of the model showed ca. 65 % loss of epithelial cells (Argasiński et al. 2012). We also know from the study by Bednarska and Laskowski (2008) that at 2400 mg Ni kg^−1^ (40.9 mM kg^−1^) the test species can live for a long time and reproduce but its respiration rate is affected, indicating some toxic effects of Ni on physiological processes. In the study on another epigeal species, *Porcellio scaber*, Donker et al. (1998) found that 40 mM Zn kg^−1^ in litter did not affect the feeding rate of the animals. Thus, we decided to use the nominal concentration of 40 mM kg^−1^ for all three metals, accepting that it is extremely high for Cd and can cause a high mortality. This turned out not to be the case, with 89 % of the Cd-treated individuals surviving until the sampling date or the end of experiment. Thus, the concentration used, although certainly representing the higher end of possible exposures, was still reasonable to achieve the aims of the study.

All three metals exhibited a complex toxicokinetic pattern with a rapid increase in internal concentrations after 1–4 days of exposure, followed by fluctuating internal concentrations during metal exposure. Such a toxicokinetic pattern, inconsistent with the classic one-compartment model, seems not so exceptional. An initial concentration increase followed by a decrease well before the animals were transferred to the uncontaminated food was observed previously for the same species, *P. oblongopunctatus*, exposed to Cd (Lagisz et al. 2005) and Ni (Bednarska et al. 2011). Also, internal Zn concentrations fluctuated over the exposure time in Zn-exposed beetles (Lagisz et al. 2005). In the centipede *Lithobius forficatus* the level of Cd increased at first and then steadily decreased even though the animals were regularly fed Cd-contaminated *Chironomus* larvae (Descamps et al. 1996). A toxicokinetic pattern different to the traditionally used two-phase one-compartment model was also found by Spurgeon and Hopkin (1999): fast initial copper accumulation was followed by a rapid excretion already during the contamination phase in Cu-exposed earthworms *Eisenia fetida*. The above-mentioned studies suggest that at least under some circumstances (e.g., particular metals, species and/or food sources), the classic two-phase one-compartment model is not adequate and, although plausible because of its simplicity, it probably omits some important physiological mechanisms shaping the toxicokinetics of metals in invertebrates when exposed to (highly) contaminated food.

The initial high increase in metal concentrations, within the first 1–4 days, followed by a concentration decrease, fulfilled our predictions based on the model by Argasinski et al. (2012). Although it was impossible to estimate exactly the percentage of damaged cells in the midgut, we showed that the damage was indeed extensive and differentiated between the metals, being particularly fast in Cd-treated beetles, more gradual in the case of Ni, and smallest in Zn-exposed animals. Cadmium exposure induced strong degenerative necrotic changes in the midgut already after 1–2 days of exposure. In turn, Ni induced swelling of cells next to the midgut lumen and their degeneration mostly by necrosis after 8 days of exposure. Longer exposure to Ni induced cell swelling and degeneration also in deeper layers of the midgut. However, in insects exposed to Ni for 28 days, after 4 days of decontamination first epithelial cells were observed. The results showed that Cd is most toxic and destroys the midgut epithelium already after short exposure at the applied concentration. The damage was profound and cells were eliminated by necrosis rather than apoptosis. It is assumed that when cell damage is too large, the apoptotic process cannot be fully realised, and cells die following the necrotic pathway (Chandra et al. 2000). In contrast to Cd and Ni, Zn was less toxic to the midgut cells, which were mostly eliminated by apoptosis, and its concentration was better regulated. Nevertheless, the progressive cell death of the gut epithelium was also observed in the beetles exposed to this metal.

The midgut epithelium of insects is composed mainly of digestive cells, responsible for the digestion and absorption of food, and regenerative stem cells, which play a role in cell renewal (Micchelli and Perrimon 2006). The midgut epithelium is the first organ exposed to excessive levels of metals ingested by an insect. Metals assimilated in excess of requirements in insects are stored by metal-binding proteins and in intracellular granules and spherites to prevent them from interfering with biochemical reactions in the tissues. When reaching their capacity for metal storage, intestinal cells break down and the accumulated metals are released into the midgut lumen and excreted (Hopkin 1989). The knowledge about cell degeneration in insect tissues and organs in response to environmental stressors is, however, scarce (Rost-Roszkowska et al. 2008). The elimination is possible throughout the processes of apoptosis, necrosis and autophagy. The first two processes are involved in the degradation of midgut epithelial cells, whereas the autophagy is mainly involved in the removal of the damaged organelles utilised by formation of vacuoles and lysosomes (Rost-Roszkowska et al. 2008). Both apoptosis and necrosis are responsible for cell elimination, but they differ in their source, course and the changes they cause. Apoptosis is an actively regulated physiological process that enables removal of useless or unexploited cells, whereas necrosis is a passive cell death resulting from acute cellular dysfunction in response to severe stress conditions or after exposure to toxic substances (Chandra et al. 2000). Under physiological conditions, apoptosis enables the balance between the rate of proliferation and elimination of destroyed or unnecessary cells (Zakeri and Lockshin 2002). Many factors, both natural and anthropogenic, including intoxication with metals, may disturb the process of apoptosis and cause enhancement of the degenerative processes in the midgut of insects (Rodrigues et al. 2008; Wilczek 2005; Wilczek et al. 2014). Even a sub-lethal level of cadmium (1.3 mM kg^−1^ dw) administered with the diet during short-term exposure (10 days) induced the programmed death of epithelial hepatopancreatic cells of the terrestrial pulmonate snail *Helix pomatia* (Chabicovsky et al. 2004), but autophagy rather than apoptosis was found by the authors. It is worth noting that the autophagy observed by Chabicovsky et al. (2004) appeared at Cd concentration in hepatopancreas of 2.2 mM kg^−1^ – similar to the equilibrium body concentration estimated for *P. oblongopunctatus* in our experiment (1.44 mM kg^−1^). However, as the authors stressed, autophagy and apoptosis should not be considered as mutually exclusive phenomena, but rather as a flexibility in a cell’s response to changes in environmental conditions. Contrary to our study, in which the exposure to Cd induced fast degeneration of the midgut by necrosis, nearly no necrotic cells were registered at any Cd concentration tested in *H. pomatia* (Chabicovsky et al. 2004). The cell loss recorded by histological analysis in *H. pomatia* was accompanied by enhanced cell proliferation (Chabicovsky et al. 2004), which was not observed in our study. Starvation and exposure to dimethoate was also shown to enhance apoptotic and necrotic changes in the midgut glands of the wolf spider *Xerolycosa nemoralis,* and the frequency of degenerative changes in starving individuals was higher than in intoxicated ones regardless of the previous pre-exposure to metals (Wilczek et al. 2014).

The exposure to Zn induced slow degenerative changes in the midgut. At the beginning of Zn exposure apoptotic cells were present in the apical part of the midgut but later also in the cells in regenerative pouches. The number of apoptotic cells increased with exposure time and they were also present in the epithelium of insects after 2 weeks of detoxification. The study on *Spodoptera litura* showed that the intensity of Zn effects on apoptosis depended on both the metal concentration in cells and the exposure time: cell ultrastructural changes in the *S. litura* midgut were closely correlated with the Zn accumulations in the midgut (Shu et al. 2012), but the apoptosis was significantly induced only at the concentration of 1000 mg kg^−1^ (15.3 mM kg^−1^) in the diet (Xia et al. 2005). In our study, however, the increased cell apoptosis in the beetles from Zn treatment could also be caused by Gregarinasine parasites observed in large numbers in the midgut cells throughout the experiment, including the detoxification phase. Indeed, in different insects with midgut cells infected by viruses or parasites, apoptosis was shown as the mechanism to eliminate cells damaged by them (Vaidyanathan and Scott 2006). In turn, lower numbers of the parasites observed in the midgut of beetles exposed to Cd or Ni might result from either the higher toxicity of these two metals to the parasites or the massive damage to the midgut and cell shedding.

One weakness of the study stems from the fact that individual beetles may differ vastly in the rate of gut damage and regeneration. The actual patterns, both in body metal concentration and midgut condition, may thus be obscured by high inter-individual variability at each sampling date. The high variance observed in metal concentrations in the beetles throughout the experiment may be a reflection of these differences in midgut cells’ turnover rates in individual beetles. Similarly, a high variation in microscopic preparations was observed. Unfortunately, this problem is hard to overcome as sampling for histopathological studies is destructive and does not allow for the following of changes in single individuals throughout the experiment.

In summary, the changes observed in the intestine during the intoxication and detoxification phases, as found in the midgut morphology at light microscope level, the apoptotic cells detected using TUNEL method, and the degenerative changes in the midgut revealed by TEM, confirm our hypothesis that heavy damage to the epithelial cells followed by their discharge to the gut lumen and excretion can be the major mechanism responsible for shaping metal assimilation to and elimination from the body. Accepting this mechanism sheds a new light on (metal) toxicokinetics, especially at high concentrations in food. This suggests that the traditional approach to (metal) toxicokinetics, based on a one-compartment exponential model, should be revised, and that the toxicokinetics should be examined thoroughly at a range of metal concentrations in food for which body concentrations are measured in parallel with studies of the histological effects in the alimentary tract.
